# A Composite Membrane of Caesium Salt of Heteropolyacids/Quaternary Diazabicyclo-Octane Polysulfone with Poly (Tetrafluoroethylene) for Intermediate Temperature Fuel Cells

**DOI:** 10.3390/membranes2030384

**Published:** 2012-07-10

**Authors:** Chenxi Xu, Xu Wang, Xu Wu, Yuancheng Cao, Keith Scott

**Affiliations:** School of Chemical Engineering & Advanced Materials, Newcastle University, Newcastle NE1 7RU, UK; Email: chenxi.xu@ncl.ac.uk (C.X.); ryanwang316@qq.com (X.W.); xu.wu@ncl.ac.uk (X.W.); yuancheng.cao@ncl.ac.uk (Y.C.)

**Keywords:** composite membrane, intermediate temperature fuel cells, heteropolyacids, microporous PTFE, polysulfone

## Abstract

Inorganic-organic composite electrolyte membranes were fabricated from Cs_X_H_3−X_PMo_12_O_40_ (CsPOMo) and quaternary diazabicyclo-octane polysulfone (QDPSU) using a polytetrafluoroethylene (PTFE) porous matrix for the application of intermediate temperature fuel cells. The CsPOMo/QDPSU/PTFE composite membrane was made proton conducting by using a relatively low phosphoric acid loading, which benefits the stability of the membrane conductivity and the mechanical strength. The casting method was used in order to build a thin and robust composite membrane. The resulting composite membrane films were characterised in terms of the elemental composition, membrane structure and morphology by EDX, FTIR and SEM. The proton conductivity of the membrane was 0.04 S cm^−1^ with a H_3_PO_4_ loading level of 1.8 PRU (amount of H_3_PO_4_ per repeat unit of polymer QDPSU). The fuel cell performance with the membrane gave a peak power density of 240 mW cm^−2^ at 150 °C and atmospheric pressure.

## 1. Introduction

Fuel cells are potential power sources for future pollution-free applications. Polymer electrolyte membrane fuel cells (PEMFCs) are an important type of fuel cell for applications in portable devices, stationary and transport power supply. Perfluorosulfonic acid membranes, such as Nafion^®^, are well-known commercial membranes for PEMFCs. However, there are issues with the use of PEMFCs such as relatively high cost (Nafion and Pt based catalysts) and operating temperature restrictions (<100 °C). These limitations have stimulated interest and commercialization in fuel cells at the intermediate temperature range of 100–400 °C [1,2]. These types of fuel cells offer several advantages such as [3,4,5,6,7]:

(1) High CO tolerance and thus the ability to use less pure hydrogen fuels,
(2) Enhancement in efficiency,
(3) Avoidance of the cathode electrode flooding by water formed in the cell reaction between hydrogen and oxygen (Equation 1):

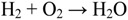
(1)
(4) Potential to use non-noble metal catalyst,
(5) System simplification through removal of humidification requirements and better cell heat transfer.

In the intermediate temperature range, several types of solid state electrolytes have been considered such as non-fluorinated polymers, pyrophosphates [8] heteropolyacids and solid acids [9]. In the lower end of this intermediate temperature range, (*i.e*., 120–200 °C) polymer electrolytes loaded with phosphoric acid (PA) have been dominant. Such materials may reduce corrosion problems and improve PA immobilisation compared to its use in phosphoric acid fuel cells (PAFCs) [7]. Polybenzimidazole (PBI) is the best known example of a membrane material used as a solid state electrolyte when loaded with phosphoric acid. Such membranes have been used for fuel cells providing high conductivity and good thermal and chemical resistance [5]. 

Based on our previous work, quaternary diazabicyclo-octane polysulfone (QDPSU) was successfully synthesised and considered as a membrane for intermediate temperature fuel cells, giving a power density of 400 mW cm^−2^ at 150 °C and atmospheric pressure [10]. Several inorganic materials, such as TiO_2_ [11], caesium substitute heteropolyacids [7] and graphite oxide [12] have been combined with PBI to form composite membranes with enhanced conductivity. 

Good mechanical strength is an important property for a membrane in fuel cells. Polytetrafluoroethylene (PTFE) is a material that can increase the mechanical strength of the membrane, as reported by Li *et al*. with PA loaded PBI [13]. Xing *et al*. reported a montmorillonite/sulfonated poly (phenylether sulfone)/PTFE composite membrane that provided good strength because of the introduction of the PTFE microporous film [14].

In many cases high loadings of phosphoric are used with PBI membranes to enhance conductivity [5]. However excess phosphoric acid, *i.e.*, more than 2 per repeating unit (PRU), may result in a reduction in mechanical strength and elution problems of electrolytes [12]. Especially above 100 °C, free acid may be lost with evaporated water which could result in instability. Ideally what is required is a membrane material that has high conductivity with a low acid loading and our approach was to use a composite of an inorganic oxide (conducting) with PBI. In this work, a Cs_X_H_3−X_PMo_12_O_40_ (CsPOMo)/quaternary diazabicyclo-octane polysulfone with polytetrafluoroethylene (QDPSU/PTFE) composite membrane was prepared with a low phosphoric acid loading to provide good proton conductivity and mechanical strength. 

## 2. Results and Discussion

**Figure 1:**
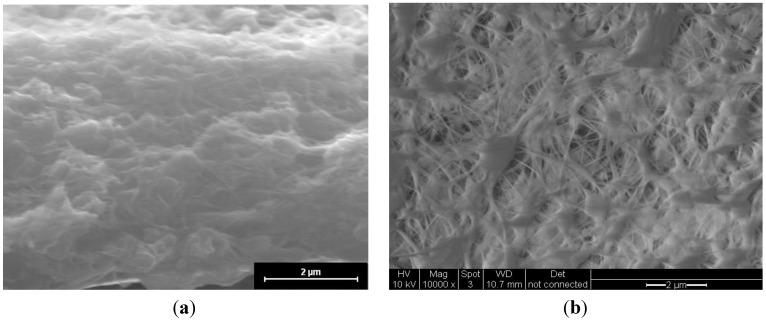
SEM of (**a**) CsPOMo/QDPSU/PTFE/H_3_PO_4_ composite membrane; and (**b**) PTFE.

**Figure 2:**
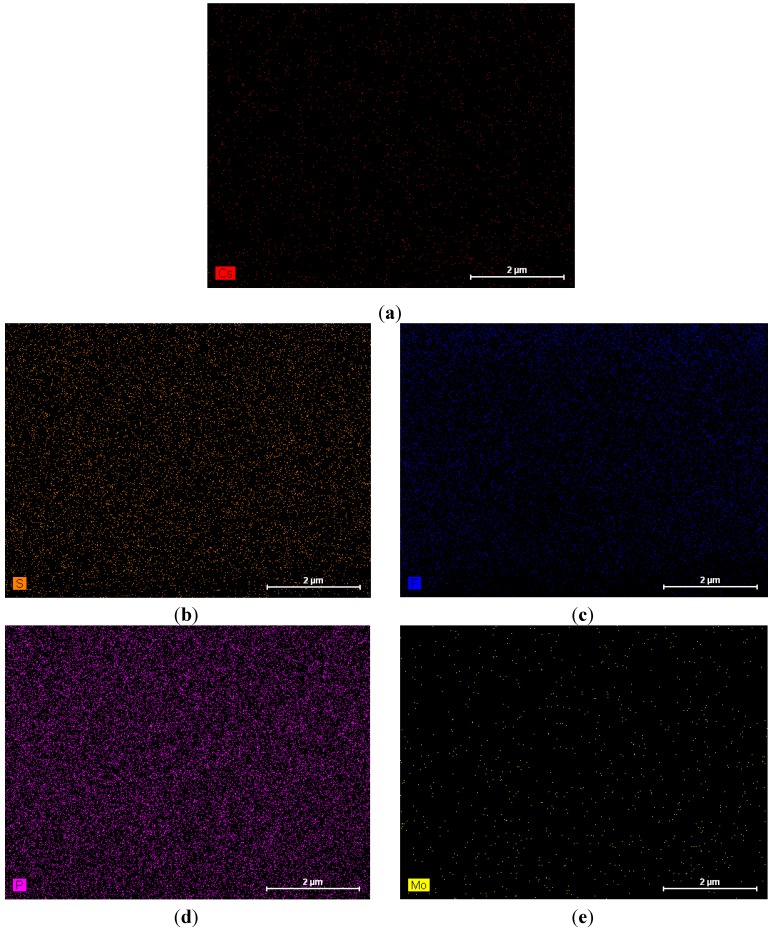
EDX analysis of CsPOMo/QDPSU/PTFE/H_3_PO_4_ composite membrane. (**a**) Cs; (**b**) S; (**c**) F; (**d**) P; (**e**) Mo.

Figure 1 shows an image of the cross-section of the CsPOMo/PSU/PTFE/H_3_PO_4_ membrane. The dense structure of the composite membrane indicates that the pores of the PTFE membrane were filled with the CsPOMo and QDPSU. The distributions of caesium, phosphorous and molybdenum elements in the membrane were analysed using EDX and are shown in Figure 2. The distribution of the CsPOMo and QDPSU is evidenced by the Cs, sulphur and fluorine elements. Phosphorous represented the H_3_PO_4_ loading in the membrane. As shown in Figure 2, a homogenous structure of the composite membrane and a good dispersion were obtained. 

**Figure 3:**
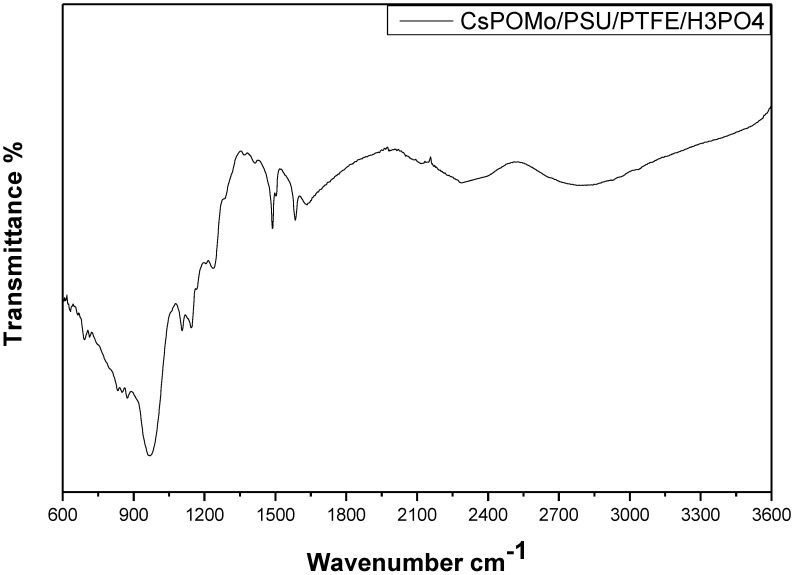
Infrared spectra of CsPOMo-PSU-PTFE-H_3_PO_4_ composite membrane.

Figure 3 shows the infrared spectra of CsPOMo-PSU-PTFE-H_3_PO_4_. The characteristic vibration bands of P=O (1,065 cm^−1^), of CsPOMo are apparent [7]. The small peaks at 2,924 cm^−1^, 1,620 cm^−1^ and the sharp peak at 1,490 cm^−1^ were attributed to quaternary ammonium group stretching vibration [10]. For the PA/QDPSU membrane, a significant vibration peak was found at 960 cm^−1^ and was attributed by the P=O vibration. The data suggest the successful preparation of CsPOMo-PSU-PTFE-H_3_PO_4_ membranes.

**Figure 4:**
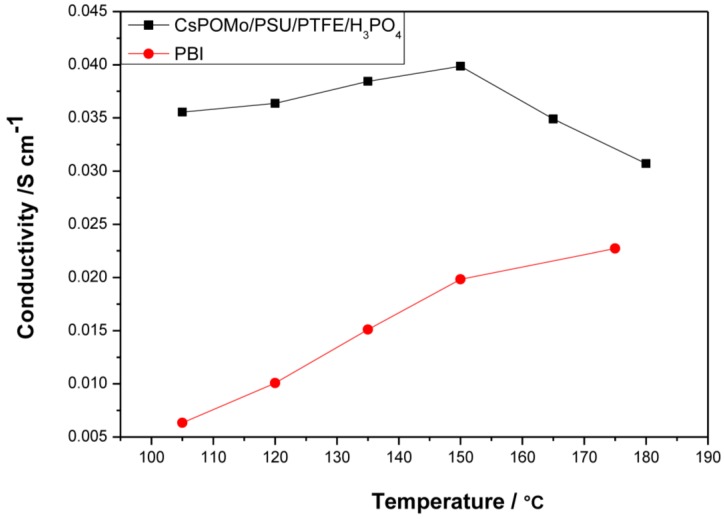
Conductivities of CsPOMo/PSU/PTFE composite membrane and polybenzimidazole (PBI) membrane loaded with H_3_PO_4_ (PRU 1.8) under relative humidity <1%. The thickness of both membranes is 30μm.

Figure 4 shows conductivity data of the CsHPA/PSU/PTFE membrane loaded with phosphoric acid (PRU 1.8) at a relative humidity <1%. The lower PA loading was chosen because excess phosphoric acid may result in a reduction of mechanical strength and elution problems of electrolytes [10]. In general the PA inside the polymer membranes would be of two types “free acid” and “bonded acid”. The amount of “bonded acid” corresponds to 2.0 molecules of PA per repeat unit of QDPSU with the nitrogen sites for hydrogen bonding. Thus PA would more likely be retained inside the membrane and less easily removed during fuel cell applications. 

Compared to the PBI membrane (0.02 S cm^−1^ at 150 °C) with the same PA loading, the composite membrane had a much higher conductivity. The conductivity of composite membrane varied from 0.035 to 0.04 S cm^−1^ in the temperature range of 105 to 150 °C. The highest conductivity of the PBI membrane was only 0.023 at 180 °C. The CsPOMo enhanced the conductivity of the membrane which may be due to a combination of the CsPOMo powder and the PSU/PTFE [7]. The combination of the hydrophobic PTFE substrate and hydrophilic (QDPSU) structure could improve water retention inside the membrane which can benefit conductivity [15]. The conductivities decreased when the temperature exceeded 150 °C, which was caused by dehydration of PA to form polyphosphate. However, this membrane could be considered for use at 150 °C. 

**Figure 5:**
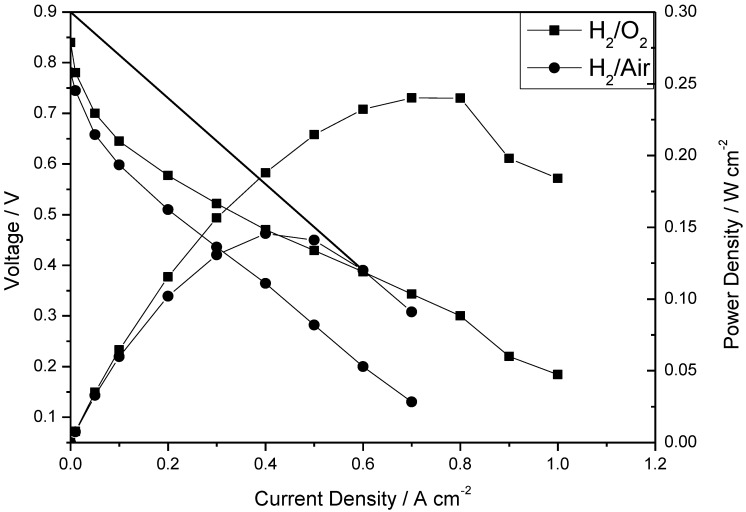
Polarization and power density curves of a fuel cell operated at 150 °C with (**a**) H_2_/O_2_; and (**b**) H_2_/air atmospheric pressure. Pt loading: cathode 0.4 mg cm^−2^; anode 0.2 mg cm^−2^; no gas humidity, H_3_PO_4_ PRU: 1.8, membrane thickness 28 μm. Gas rate: anode: 40 dm^3^ min^−1^; cathode: 70 dm^3^ min^−1^.

**Figure 6:**
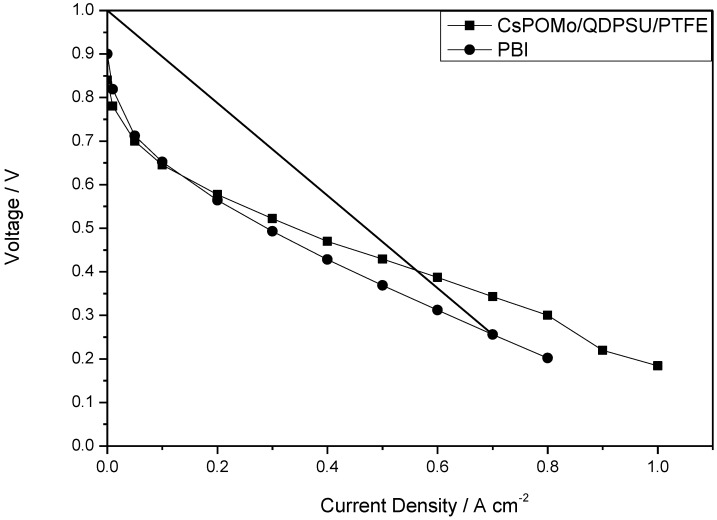
Polarization curves of CsPOMo/QDPSU/PTFE and PBI fuel cell operated at 150 °C with H_2_/O_2_. Atmospheric pressure, no gas humidity.

The polarization and power density curves of the H_2_/O_2_ fuel cell obtained at 150 °C at low humidity (<1%) with the CsPOMo/PSU/PTFE membrane are shown in Figure 5. The total Pt catalyst loading was relatively low at 0.6 mg cm^−2^. The cell open circuit voltage was 0.85 V, which indicates that the relatively thin membrane (28 μm) was partially gas permeable, which probably resulted in some gas crossover and thus electrode polarization caused by mixed potentials at the electrodes. However, the thin membrane provided a reasonably good conductivity. A peak power density of 240 mW cm^−2^ was achieved with oxygen. This peak power was only slightly better than that with a low acid loading PBI membrane (220 mW cm^−2^, doping level 1.9) used in our previous work as shown in Figure 6 [11]. The performance achieved by the composite membrane could be attributed to its greater proton conductivity and superior water retention properties on the membrane surface at low acid loading [7,11].

The fuel cell with hydrogen and CO_2_ free air gave a peak power density of 140 mW cm^−2^ which was lower than that with oxygen, as would be expected from the lower partial pressure of oxygen with the former. 

The internal resistance, as estimated from the voltage drop in the intermediate voltage range, gave a cell conductivity of around 0.006 S cm^−1^. This conductivity was much less than that of the membrane itself and indicates significant voltage loss in the electrode layers (and other cell components). Furthermore, as Figure 5 shows, there was an approximate 200 mV voltage loss from current densities of 0 to 0.1 A cm^−2^ indicating that the catalyst compositions in the MEA were not “optimal” for the fuel cell. As no PA electrolyte was added to the catalyst layers and in theory no mobile PA would be present from the membrane, due to the low PA loading used, the electrode layer would have a relatively low ionic conductivity and thus catalyst utilization in the electrode reactions would be relatively low. Essentially only the Pt catalyst next to the membrane would be active, consequently explaining the high electrode activation losses. 

**Figure 7:**
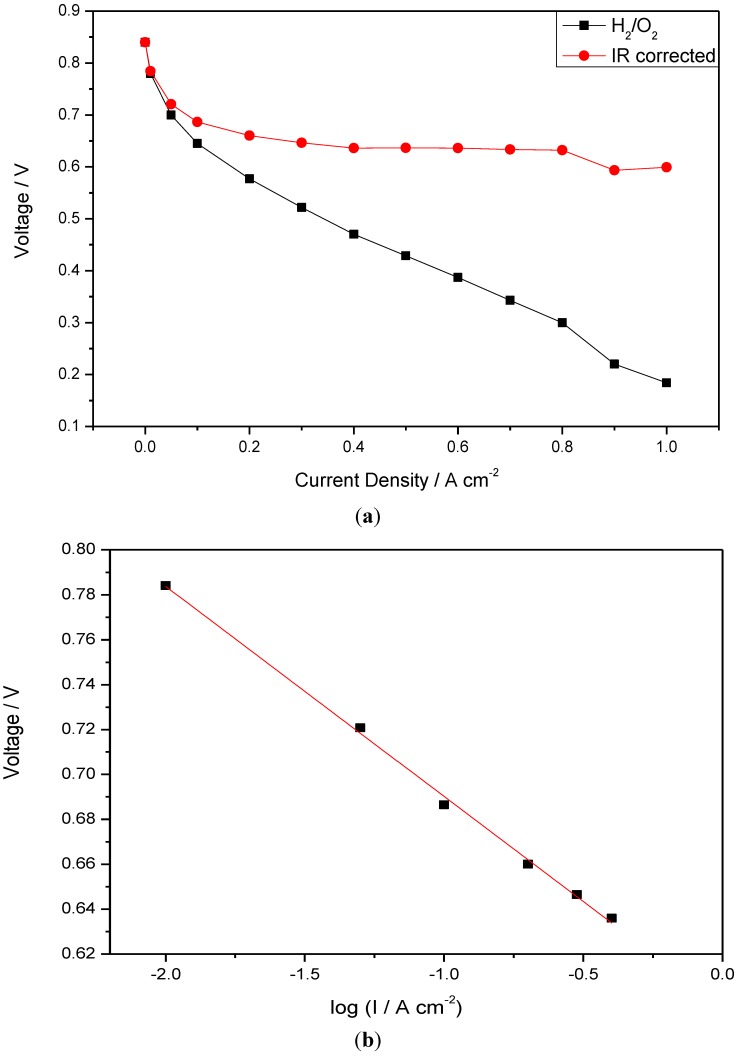
(**a**) IR corrected polarization curves of CsPOMo/PSU/PTFE; (**b**) Tafel plots obtained from polarization curves (I is current density).

Figure 7 shows IR corrected polarization curves of the fuel cells operated with H_2_/O_2_ at 150 °C. IR correction is based on the combined resistance of membrane and electrode, measured from the slope of the cell voltage *vs*. current density at the high current density region (0.4–0.8 A cm^−2^). In this region the voltage loss associated with electrode polarization is small compared to that of the internal resistance. From the plot of the IR corrected voltage *vs*. log (current density I), the slope (Tafel type) of the line is approximately 98 mV per decade. The value is close to the literature reported values (100 mV dec^−1^) on ORR electrodes for phosphoric acid loaded PBI fuel cells [16]. 

Overall, although the CsPOMo/PSU/PTFE composite is a potential membrane for polymer electrolyte membrane fuel cells, more studies are required to investigate the electrode catalyst layer composition and to establish suitable MEA preparation. Ideally this should incorporate the membrane material in the catalyst layers, and is the subject of on-going research.

## 3. Experimental

### 3.1. Composite Membrane Preparation

Cs_X_H_3−X_PMo_12_O_40_ was prepared according to published procedures [7]. 0.407 g Cs_2_CO_3_ (Aldrich) dissolved in 10 cm^3^ (mL) deionised water was added to a solution of 2.88 g H_3_PMo_12_O_40_ (Aldrich) dissolved in 30 mL deionised water to achieve the mole ratio of Cs_2_CO_3_:H_3_PMo_12_O_40_ of 1.25:1. The solution was evaporated at 60 °C for 24 h and rinsed in de-ionised water three times [7]. 

QDPSU was made as described in previous work [12]. Briefly, the choloromethylated polysulfone (CM-PSU) was synthesised through the Friedel-Crafts reaction. 10 g polysulfone (P-1700, Udel) was dissolved in 400 mL 1,2-dichloroethane. 6.78 g paraformaldehyde and 24.6 g trimethylchlorosilane were added to the solution with magnetic stirring. Stannic chloride 1.178 g was added drop-wise and the reaction was carried out for 12 h at 50 °C. The CM-PSU was formed as a white precipitate in ethanol (99.9%) to remove the excess reactants. The CM-PSU and 1,4-diazabicyclo[2.2.2]octane (DABCO) was mixed in DMAc in a molar ratio of 1:5, with vigorous stirring at 80 °C for 14 h, whereupon the final product was precipitated using diethyl ether and dried in a vacuum. CsPOMo was dissolved in QDPSU DMAc solution at a weight ratio of 30 wt % to form the resultant 

The PTFE membrane used in this work was obtained from Membrane Solution (New York, NY, USA). It was 25 μm in thickness and had a porosity of 82% and a pore size between 0.2 and 0.3 μm. The hydrophobic PTFE sheet was pre-treated, to enable incorporation of the QDPSU polymer solution, using the method of Li *et al*. [13] which involved treatment with seven parts H_2_SO_4_ (98 wt % aqueous solution Aldrich) and three parts H_2_O_2_ (30 wt % aqueous solution Aldrich) at 80 °C for 1 h. Following this, the porous PTFE was rinsed with copious amounts of deionised water and further treated by immersion in a solution containing one part aqueous NaOH (1.0 mol dm^−3^) solution, one part H_2_O_2_ (30 wt % aqueous solution), and five parts deionised water at 70 °C for 30 min, followed by rinsing with copious amounts of DI water. The pre-treated PTFE sheet was boiled in DMAc for 10 min, and then immersed in 3 wt % CsPOMo/QDPSU solution, for 10 min and placed on an optical glass plate and left for 6 h at 60 °C to form the CsPOM/QDPSU/PTFE membrane. 

The QDPSU polymer with a degree of substitution of 106% (1.06 DABCO units in each unit of PSU molecule) was used for the preparation of composite membrane. The membrane was immersed in 2.0 M H_3_PO_4_ at room temperature for 3 days [8,10].The theoretical amount of “bonded acid” corresponded to 2.0 molecules of PA per repeat unit of QDPSU with nitrogen sites for hydrogen bonding. Thus with a 2.0 M PA, the acid content will be less and was measured at 1.8 PRU. The data in this work was compared against a PBI membrane (30 μm in thickness) which was prepared by a casting method as described previously [13].

### 3.2. Membrane Characterisation

The in-plane conductivity of the membranes was measured using a four-point probe method with a frequency response analyzer (Voltech TF2000, Abingdon, UK). A rectangular membrane sample (10 mm by 50 mm) was placed across four platinum foils (probes) with equal spacing of 5 mm. AC impedance measurements were carried out between frequencies of 1 and 20 kHz. The membranes were held at anhydrous conditions and atmospheric pressure for 2 h before testing, and measurements were taken at 30 min intervals to ensure the membrane reached a steady state.

The crystal structures of different powders were analysed by X-ray diffraction (XRD, PANalytical X’Pert Pro Diffractometer), with a scan range of 5–70° over 2 h. The morphologies of membranes were investigated by a JSM-5300LV (JEOL, Tokyo, Japan) Scanning Electron Microscope (SEM). Fourier transform infrared spectroscopy (FTIR) of different samples was performed with a Varian 800 FT-IR spectrometer system between 4,000 and 400 cm^–1^.

### 3.3. Membrane Electrode Assemblies (MEA)

Gas diffusion electrodes (carbon paper) incorporated with wet proofed micro-porous layer (H2315 T10AC1), obtained from Freudenburg (FFCCT, Weinheim, Germany), were used as substrates to deposit the catalyst layer for both anode and cathode. Catalyst inks were prepared by blending carbon-supported catalysts (50 wt % Pt/C, Alfa Aesar) and polytetrafluoro-ethylene (PTFE) solution (60 wt % Aldrich) in a water–ethanol mixture under ultrasonic vibration for 10 mins [12,13]. The catalyst inks were sprayed onto the micro-porous gas diffusion layer, at 100 °C, to achieve Pt loading of 0.6 mg cm^−2^. The MEA was finally obtained by hot pressing at 150 °C and 40 kg cm^−2^ pressure for 10 min.

### 3.4. Fuel Cell Tests

The MEA was fixed between two high-density graphite blocks (impregnated with phenolic resin) with parallel gas flow channels. The active electrode area was 1 cm^2^. Electric cartridge heaters were mounted at the rear of the graphite blocks to maintain the desired temperature. H_2_ and O_2_ or air were fed to the cell at flow rates of 0.4 and 0.7 dm^3^ min^−1^ respectively. 

## 4. Conclusions

A thin CsPOMo-PTFE-QDPSU composite membrane was prepared and characterized for use in a high temperature PEMFC. The ionic conductivity of the composite membrane, loaded with a low content of H_3_PO_4_, was 0.04 S cm^−1^ at 150 °C which was twice that of the pristine loaded PBI (0.02 S cm^−1^) with the same PA doping level. In fuel cell tests, power densities of 240 mW cm^−2^ and 140 mW cm^−2^ were achieved using oxygen and air respectively. The data indicate that the CsPOMo-PTFE-QDPSU composite can be considered as a candidate membrane for high temperature PEMFC because of its acceptable conductivity and fuel cell performance with a low PA acid loading. More studies are required to optimize the electrode catalyst layer composition and to establish suitable MEA preparation.
